# Analysis of STAT3 post-translational modifications (PTMs) in human prostate cancer with different Gleason Score

**DOI:** 10.18632/oncotarget.17245

**Published:** 2017-04-19

**Authors:** Rossana Cocchiola, Donatella Romaniello, Caterina Grillo, Fabio Altieri, Marcello Liberti, Fabio Massimo Magliocca, Silvia Chichiarelli, Ilaria Marrocco, Giuseppe Borgoni, Giacomo Perugia, Margherita Eufemi

**Affiliations:** ^1^ Department of Biochemical Sciences, Sapienza University of Rome, Rome, Italy; ^2^ Istituto Pasteur, Fondazione Cenci Bolognetti, Piazzale Aldo Moro 5, Rome, Italy; ^3^ Department of Gynecological-Obstretic Science and Urologic Sciences, Sapienza University of Rome, Rome, Italy; ^4^ Department of Radiological, Oncological and Pathological Sciences, Sapienza University of Rome, Rome, Italy; ^5^ Fondazione Enrico ed Enrica Sovena, Rome, Italy

**Keywords:** STAT3, PTMs, signaling pathways, prostate cancer, biomarkers

## Abstract

Prostate Cancer (PCa) is a complex and heterogeneous disease. The androgen receptor (AR) and the signal transducer and activator of transcription 3 (STAT3) could be effective targets for PCa therapy. STAT3, a cytoplasmatic latent transcription factor, is a hub protein for several oncogenic signalling pathways and up-regulates the expression of numerous genes involved in tumor cell proliferation, angiogenesis, metastasis and cell survival. STAT3 activity can be modulated by several Post-Translational Modifications (PTMs) which reflect particular cell conditions and may be implicated in PCa development and progression. The aim of this work was to analyze STAT3 PTMs at different tumor stages and their relationship with STAT3 cellular functions. For this purpose, sixty-five prostatectomy, Formalin-fixed paraffin-embedded (FFPE) specimens, classified with different Gleason Scores, were subjected to immunoblotting, immunofluorescence staining and RT-PCR analysis. All experiments were carried out in matched non-neoplastic and neoplastic tissues. Data obtained showed different STAT3 PTMs profiles among the analyzed tumor grades which correlate with differences in the amount and distribution of specific STAT3 interactors as well as the expression of STAT3 target genes. These results highlight the importance of PTMs as an additional biomarker for the exactly evaluation of the PCa stage and the optimal treatment of this disease.

## INTRODUCTION

Prostate Cancer (PCa) is worldwide one of the most common male tumors. About 95% of prostate cancers are adenocarcinoma. PCa is characterized by an evident clinical, histological and biological heterogeneity. Clinically PCa goes from “latent” to an “aggressive” form. The “latent” form, a slow-growing tumor, appears histologically differentiated and indolent, without any symptoms. On the contrary, the aggressive form is a fast-growing tumor with a lethal progression [1, 2]. The identification of the PCa type is an important step for the optimal treatment and for the decrease of the morbidity and mortality incidence. Recently, the early stage detection is based mostly on the evaluation of the Prostate Specific Antigen (PSA) serum levels in patients. However, the most important limitation of PSA is its poor specificity. PSA concentration can be elevated also in non-malignant conditions such as prostatitis and benign prostatic hypertrophy [3]. Actually, all the parameters used in urology, PSA levels, Gleason Score classification and TNM system, are not sufficient to predict the tumor progression types [4]. Therefore, the clinicians and researchers are interested in finding more precise and sensitive biomarkers suitable for PCa diagnosis as well as prognosis and therapy. In this scenario, many data suggest a crucial role of cytokines and growth factor pathways in prostate cancer progression and in the development of therapy resistance [5–8]. A key central player of these signaling pathways is the protein Signal Transducer and Activator of Transcription 3 (STAT3) which was found constitutively activated in many cancer types including PCa [9].

STAT3 is ubiquitously expressed and plays multiple functions in normal cell [10]. In the canonical way, the latent cytoplasmic STAT3 is activated following phosphorylation of the tyrosine 705 (pY705-STAT3), in the C-terminal domain. This modification causes a conformational change in the dimers from anti-parallel to parallel and the activated STAT3 dimers translocate to the nuclear compartment [11, 12, 13]. Once in the nucleus, STAT3 regulate transcription through the binding to specific DNA response elements in the promoter regions of many target genes and moreover the nucleus can be considered to be its inactivation compartment, since STAT3 dephosphorylation at pY705 occurs mainly here [14, 15, 16]. However, recently a plethora of evidences on non-canonical STAT3 activities has emerged [17]. Severals studies suggested the presence of Stat3 in mitochondria (mitoStat3), where it acts as regulator of mitochondrial respiration and transcriptional process [18, 19] by altering ROS production. Another STAT3 non canonical activity involves unphosphorylated Stat3 (uStat3), which can also act as a nuclear transcription factor, broadening in this way the potential mechanisms involved in Stat3 signaling. These studies suggested that non-canonical Stat3 signaling, together with the well characterized, canonical Stat3 signaling pathways, may play an important role in tumor development and progression.

In addition to tyrosine phosphorylation, the STAT3 activity can be modulated by other important post-translational modifications (PTMs) [20]. The phosphorylation of Ser727 (pS^727^-STAT3) increases STAT3 transcriptional activity [21, 22] and it is necessary for her mitochondrial activities [23]. The Acetylation of Lys685 (acK^685^-STAT3), usually presents during inflammatory processes, has been reported to stabilize STAT3 dimers [24, 25]. The S-glutathionylation has been observed in response to oxidative stress [26]. Therefore, STAT3 PTMs are specific and reflect particular cell conditions and they may be implicated in PCa development and progression. Our efforts are focused on understanding the correlation between STAT3 PTMs, as well as STAT3 protein interactors and gene expression at different clinical tumor stages [27]. The protein interactors analyzed are: CBP/p300 (Histone Acetyltransferase p300 interactor), directly responsible for STAT3 acetylation [28], PDIA3/ERp57 [29] and APE1/Ref-1 [30] modulators of oxidative stress conditions. Studies were carried out using Formalin Fixed and Paraffin Embedded (FFPE) tissues, derived from radical prostatectomy of 65 patients ranging from Gleason Score 6 and Gleason Score 9. FFPE tissues are widely used as an “archival FFPE tissue banks”, since can be easily stored for years due to their inherent stability at room temperature and they have the advantage of a known patient outcome history. Therefore, these samples are invaluable source of biological information to elucidate pathological pathways or to retrieve disease-associated biomarkers.

## RESULTS

### Classification of analyzed PCa tissues

All human tissue samples were obtained from PCa patients in the “Policlinico Umberto I” Hospital of Sapienza University. The patient profiles including age, PSA value, Gleason Score and TNM system, were summarized in Table 1. Histological diagnosis was performed by independent certified pathologists of the hospital. Cancer tissues of each Gleason pattern and adjacent normal counterparts were collected separately from the formalin-fixed paraffin embedded (FFPE) sections using microtome. As a reference haematoxylin/eosin stained [31] sections of the tissues were histological verified by an experienced pathologist.

**Table 1:** Characteristic of patients classified according to Gleason Score

Groups	Gleason6	Gleason7	Gleason8	Gleason9
Age, median (range)	68, 66	62, 5	64	62, 5
Preoperative PSA (ng/ml), median (range)	5, 55	11, 36	12, 4	8, 2
pTNM				
pT3	9	3	8	1
pT4	6	12	14	12
**Total**	**15**	**15**	**22**	**13**

Note: Patient profiles including median age, median PSA value, Gleason Score and TNM system.

### Immunofluorescence analysis of pY^705^-STAT3 expression in prostate cancer tissues

**Figure 1:**
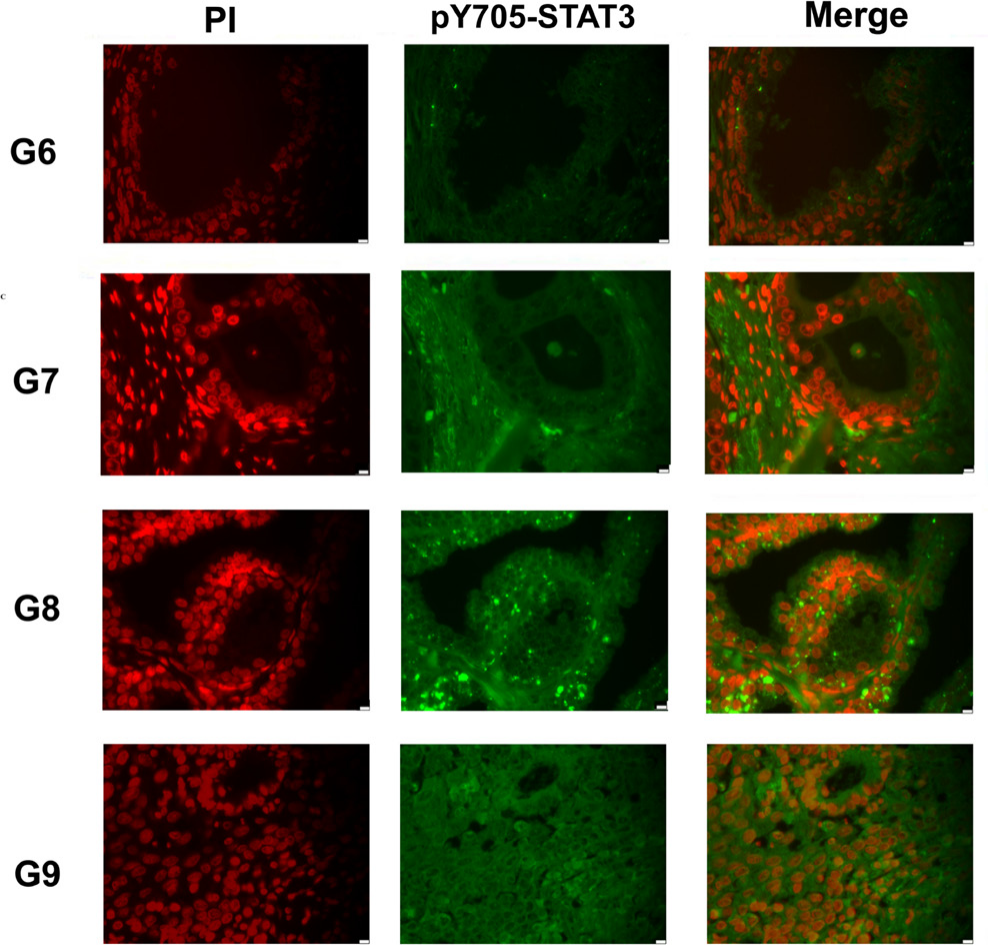
Analysis of pY^705^-STAT3 distribution in prostate cancer FFPE tissues Immunofluorescence staining of representative FFPE tissue sections with different Gleason score pY^705^-STAT3: phosphorylated at tyrosine 705, PI: Propidium Iodide, G: Gleason Score, FFPE: Formalin Fixed and Paraffin Embedded.

Sections of sixty-five FFPE of non-neoplastic and neoplastic tissue specimens were processed for immunofluorescence staining to detect STAT3 and pY^705^-STAT3, according to previously established protocols [32]. This analysis revealed a similar level of pY^705^-STAT3 modification in all tumor samples as showed in a representative image of different specimens corresponding to a Gleason score ranging from 6 to 9 (Figure 1). In 6–7 Gleason the nuclear compartment was enriched of pY^705^-STAT3. However into 8–9 Gleason we observed also an evident cytoplasmatic presence of pY^705^-STAT3; in particular this result was directly related to the persistently activated STAT3 which plays an important role in tumor progression and malignancy [16]. As negative control, sections were stained with only secondary antibodies or with primary antibodies preincubated with different concentration of a synthetic phosphopeptide corresponding to residues flanking Tyr705 of STAT3 [33]. Results were reported in Supplementary Figure 3.

### STAT3 PTMs evaluation in PCa tissues by Western blotting analysis

**Figure 2:**
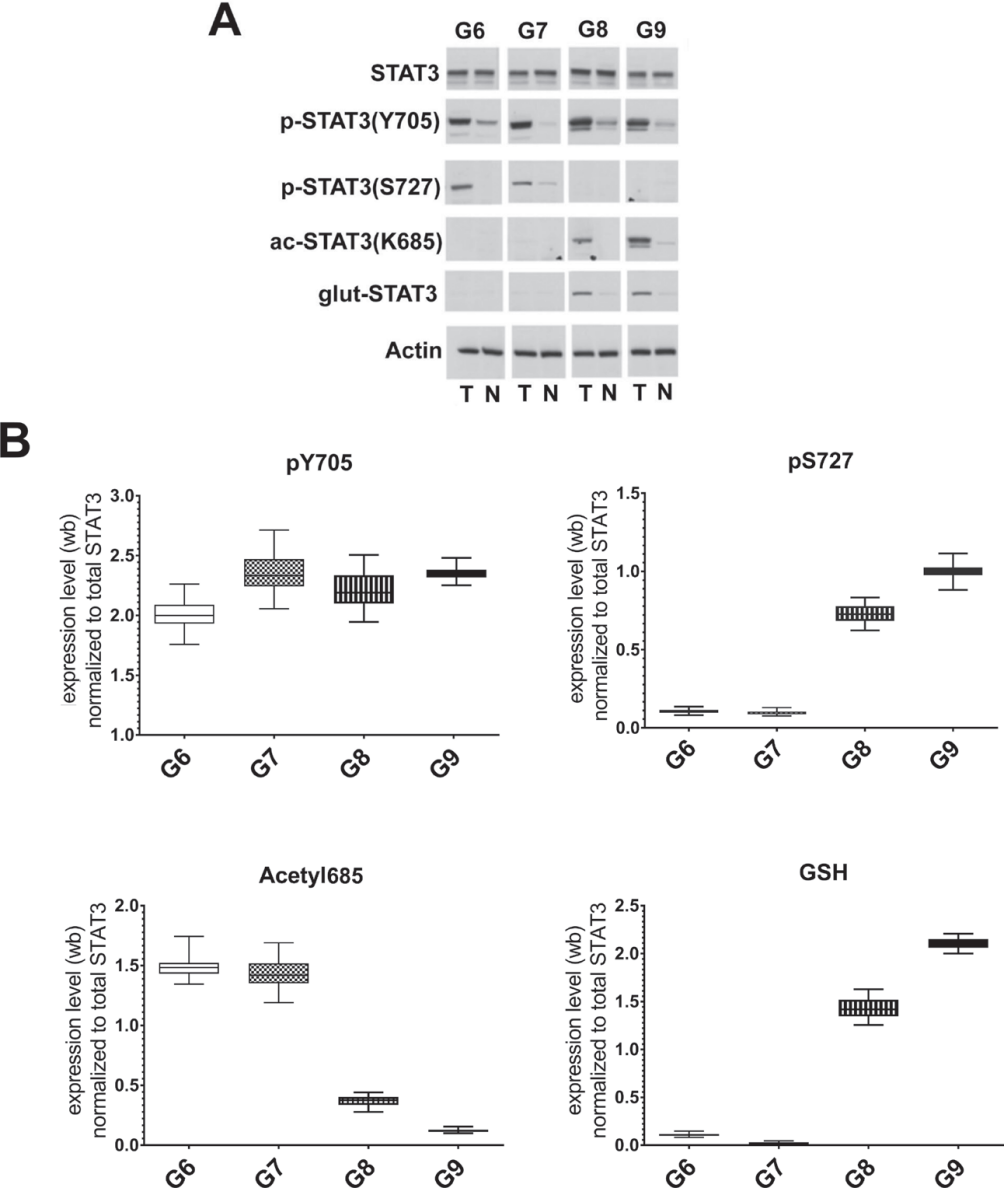
Analysis of STAT3 PTMs in FFPE samples with different Gleason score (**A**) Representative western blot analysis of protein extracted from FFPE corresponding to Gleason Scores (G) 6, 7, 8 and 9. T: prostate carcinomas; N: normal tissue, adjacent to tumor. (**B**) Cumulative results of STAT3 PTMs levels in tumor prostate tissues from 65 matched prostate carcinomas. Values are presented as the means, standard deviations (boxes) and min/max values (bars). Data were analyzed by One Way Anova test and all differences between Gleason score groups in each data set were statistically significant (*p* < 0,01%) with the exception of pS727 G6 vs G7. pY^705^-STAT3: phosphorylated at tyrosine 705, pS^727^-STAT3: phosphorylated at serine 727, acK^685^-STAT3: acetylated at lysine 685, glut-STAT3 or GSH: glutathionylated STAT3.

The analyzed FFPE tissues had different Gleason score values. Each tumor section was compared to the normal tissue adjacent to the tumor. The number of specimens for each Gleason grade is summarized in Table 1. The levels of STAT3 and its main PTMs, were detected by immunoblotting analysis with anti-STAT3, anti-pY^705^-STAT3, anti-pS^727^-STAT3 and anti-acK^685^-STAT3 antibodies. As described by Robertson et al. [32], for the glutathionylation we performed a double staining with a monoclonal antibody against protein-bound GSH and anti-STAT3 antibody. The results showed a constitutively activated STAT3 (pY^705^-STAT3) in all tumor grades compared to the matched normal sections. A significant change between the levels of the acetylated and glutathionylated forms was present in the different clinical stages. In samples with lower Gleason Score (Gleason 6 and 7), the acK^685^-STAT3 is overexpressed compared to the more malignant scores. On the other hand, the pS^727^-STAT3 and the glutathionylated forms were relevant in higher Gleason grades. Intensity of the immunostained bands was normalized to β-actin or to the total amount of proteins in the gel revealed by coomassie staining using the ImageJ program (Figure 2). The analysis of STAT3 PTM's profile was also performed on fresh tumoral and normal tissues samples from biopsies. Immunoblotting releaved similar STAT3 PTM's profiles to those obtained from FFPE samples (Supplementary Figure 2), supporting the efficiency and accuracy of the data obtained from the FFPE sections.

### Immunoblotting analysis of STAT3 interactors in PCA FFPE tissues: PDIA3/ERp57, CBP/p300 and APE1/REF-1

**Figure 3:**
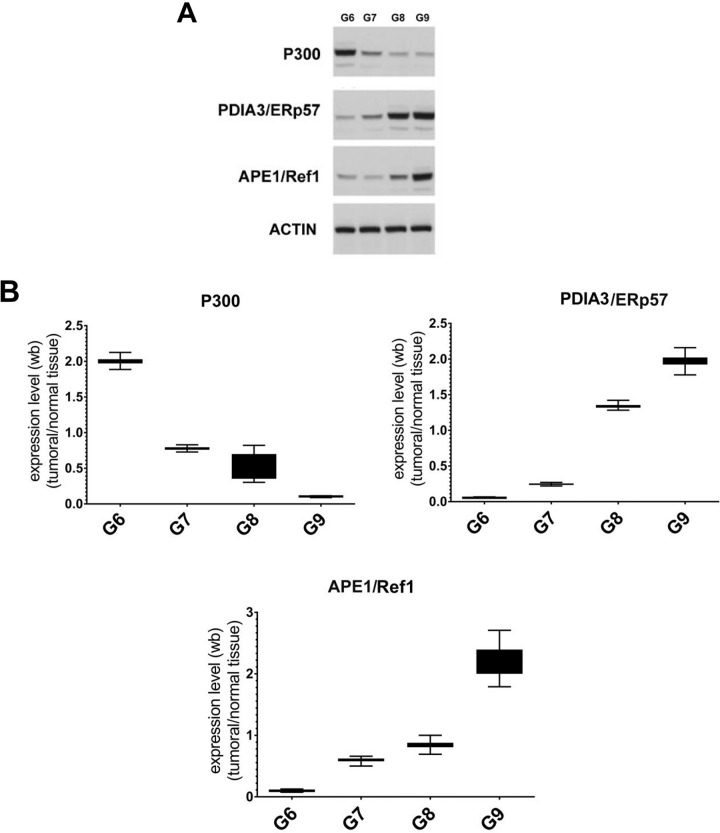
Analysis of STAT3 interactors in FFPE samples with different Gleason score (**A**) Representative Immunoblotting of selected STAT3 protein interactors expressed in each Gleason Score. (**B**) Average level of expression of the selected STAT3 protein interactors matching to the same Gleason Score normalized to the corresponding normal samples. Values are presented as the means, standard deviations (boxes) and min/max values (bars). Data were analyzed by One Way Anova test and all differences between Gleason score groups in each data set were statistically significant (*p* < 0,01%). CBP/p300: CREB-binding protein/Histone Acetyltransferase p300 interactor. PDIA3/ERp57: Protein Disulfide-isomerase A3/Endoplasmic Reticulum Protein 57, APE1/Ref-1: Apurinic/apyrimidinic endonuclease 1/redox factor-1.

We investigated the presence and relative amount of selected STAT3 interactors, CBP/p300, APE1/Ref-1 and PDIA3/ERp57 analyzing, by immunoblotting, the protein extracts derived from patients presented in Table 1. The results showed a significant expression of the protein CBP/p300 in the lower Gleason Score (Gleason 6); on the contrary, the protein APE1/Ref-1 and PDIA3/ERp57 were overexpressed in the more malignant scores (Gleason 8 and 9 (Figure 3)). Alterations in the amount of STAT3 interactors in the analyzed samples suggest a different behaviour of STAT3 which activity could correlate with the cancer progression in the tissue.

### Expression of *BIRC5, SOD2, SRD5A2 MMP2, PSA and CRP* genes correlated to Gleason Score

**Figure 4:**
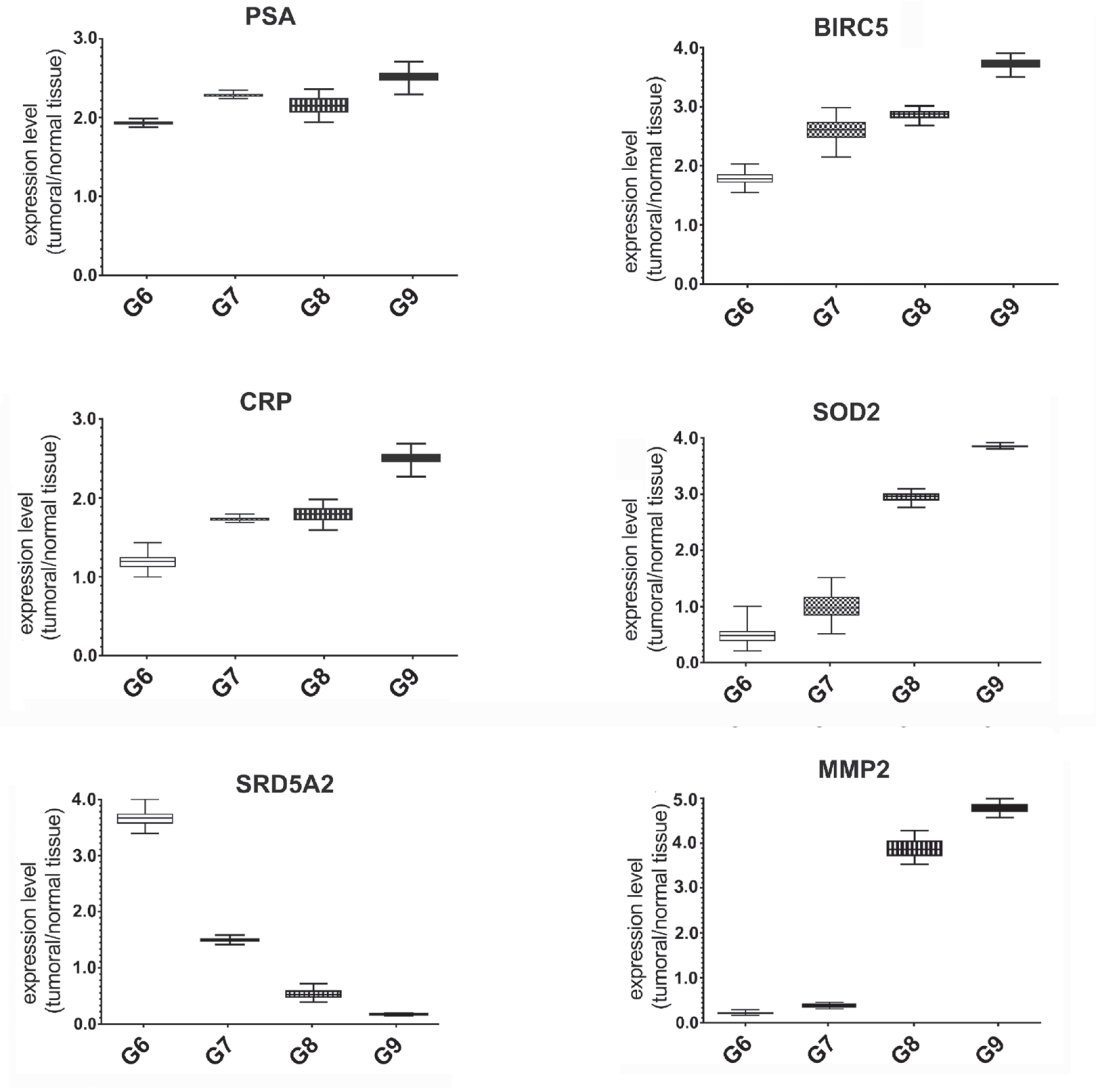
Expression analysis of STAT3 dependent genes in FFPE samples with different Gleason score Quantitative RealTime-PCR for *PSA*, *BIRC5*, *CRP*, *SOD2*, *SRD5A2* and *MMP2* genes in FFPE tissues with different Gleason Score. Each gene expression value was normalized to housekeeping gene (β-actin) and to a control sample (normal matching tissue). Values are presented as the means, standard deviations (boxes) and min/max values (bars). Data were analyzed by One Way Anova test and all differences between Gleason score groups in each data set were statistically significant (*p* < 0,01%). PSA: Prostate Specific Antigen, *BIRC5*: Baculoviral Inhibitor of Apoptosis Repeat-containing 5, *CRP*: C-reactive Protein, *SOD2*: Superoxide Dismutase 2, *SRD5A2*: Steroid-5-Alpha-Reductase, Alpha Polypeptide 2, MMP2: Matrix Metalloproteinase-2.

We evaluated the expression of several STAT3 target genes by Quantitative Real Time-PCR in all samples. In particular, we focused attention to the levels of *BIRC5, PSA, SRD5A2, MMP2 CRP* and *SOD2* genes, known to be involved in PCa initiation and progression [38–41], and associated with inflammatory [42] and oxidative stress conditions. The results showed an increase of the *MMP2*, *BIRC5, CRP* and *SOD2* expression in samples characterized by high Gleason Score. On the contrary, as described in literature [43, 44], we saw a sharp decrease of the *SRD5A2* expression in Gleason Score 9. The trend of *PSA* expression levels was variable among the different tissues (Figure 4).

## DISCUSSION

Emerging evidences revealed that cancer progression is largely regulated by protein PTMs [45, 46]. In particular, in molecular hub proteins for signaling pathways, like STAT3, PTMs may influence gene regulation, cellular functions, tissue development, diseases, malignant progression and drug resistance [47, 48]. Here, we investigated the specificity of STAT3 PTMs in different PCa Gleason Score tissues and their influences in gene expressions. STAT3, activated by phosphorylation on tyrosine 705, was shown to be associated with the malignant transformation of prostatic epithelial cells [49]. However, beside tyrosine 705 phosphorylation, other post-translational modifications have been proven to be essential for the pleiotropic functions of STAT3 [50]. Among these, acetylation of Lysine 685 by the mammalian histone acetyltransferase complex CBP/p300, has been reported to increase the transcriptional activity stabilizing STAT3 homodimers. In addition this PTM is known to be involved in inflammatory processes activated by cytokines [24, 51]. pS^727^-STAT3 is known to influence the protein through canonical and non canonical mechanisms of activation [52]. In the canonical way, which first requires tyrosine phosphorylation, pS^727^-STAT3 induces the maximal transcriptional activity of the protein [53]. In the non canonical way, STAT3 phosphorylated only on Serine 727 residue regulates its mithocondrial translocation [23]. Mithocondrial STAT3 interacts with respiratory complexes I, II, and V of the electron transport chain and influences ROS production, thus promoting tumor cell survival and invasion [54]. Several reports have highlighted that STAT3 is also a redox-sensitive protein and may be controlled by S-glutathionylation, a post-translational protein modification regulated by intracellular redox state [26, 55]. Literature data demonstrated that an increase in glutathionylated proteins is always associated with oxidative stress *in vitro* and *in vivo* [56]. The identification of PTMs involved in STAT3 regulation will also include the characterization of proteins correlated with this cellular processes, such as writers, readers, modifiers and erasers. In particular, during oxidative stress conditions, some STAT3 protein partners, such as PDIA3/ERp57 and APE1/Ref-1, are highly expressed. Other researchers confirmed the presence of these two proteins in prostate cancer [57, 58] and it has been reported an interaction between them [59]. The protein ERp57, also known as GRP58/PDIA3, is a stress-responsive protein and is a member of the protein disulfide isomerase family. It has been recognized as a multifunctional chaperone that regulates proper folding and the quality control of glycoproteins. Although PDIA3/ERp57 has been characterized by its functions in the endoplasmic reticulum (ER), many evidences indicate that PDIA3/ERp57 is also involved in a variety of functions in the cytosol and nucleus. Several reports suggested that PDIA3/ERp57 is associated with tumor progression and the modulation of STAT3 activity [60, 61]. Apurinic/apyrimidinic endonuclease/redox factor-1 (APE1/Ref-1) is a multifunctional protein that, in addition to its base excision DNA repair activity, exerts a redox control on cancer-associated transcription factors, including STAT3 [62]. Our studies, carried out on sixty-five FFPE tissues, confirmed the constitutively activation of STAT3 protein (pY^705^-STAT3) in tumor samples, without any relevant variation in expression levels between the Gleason grades. On the contrary, there is a significant increase of the acK^685^-STAT3 in the low Gleason Scores. The result was correlated with the higher presence of the histone acetyltransferase CBP/p300 interactor in these tissues. On the other hand, pS^727^-STAT3 and the glutathionylated-STAT3 forms were highly expressed in the most malignant tumor (Gleason 8 and 9) together with an increase in the APE1/Ref-1 and PDIA3/ERp57 levels which are known to be present under oxidative stress [63]. Quantitative Real Time-PCR in FFPE tissues confirmed our hypothesis. In this experiment we looked at the expression of specific STAT3 target genes involved in PCa. The *PSA* gene, as expected, was always overexpressed between the different tissue grades and its values were a reflection of the pY^705^-STAT3 levels, sustaining the fact that its expression doesn't follow an universal value, but it is variable and differs among patients. This observation, according to the data reported in the Table 1, highlights the absence of correlation between *PSA* values and the Gleason Score. *SOD2* gene expression gradually increased from low to high Gleason Scores, responding to an oxidative state [64]. Similarly, *BIRC5*, an anti-apoptotic protein, and *CRP* gene, known to be involved in response to inflammation [36], rise its expression with increasing Gleason score, as reflection of clinical-pathological patient conditions. These findings are correlated with the overexpression of the pS^727^-STAT3 in the aggressive forms, as responsible modification for the mitochondrial translocation and for the equilibrium of the ROS species. The *SRD5A2* gene expression profile showed a downregulation in Gleason Score 9, related to the progression of an aggressive hormone-independent form. The *MMP2* gene expression followed exactly the opposite trend of *SRD5A2*, and this was consistent with data described also by other researches, where exogenous expression of *SRD5A2* reduces the cell migration/invasion in cancer cells. In conclusion, and in agreement with the literature, tumor microenvironment can modify different cellular events and promote tumor progression. STAT3, as a signal mediator protein, reflects these particular tumor stages through its PTMs. Here, we demonstrated that inflammatory processes are predominant in low Gleason grades as confirmed by the presence of STAT3 acetylated and CBP/p300 interactor [65]. This modification seems related to the regulation of *MMP2* and *SRD5A2* gene expression; Sestito et al. [66] demonstrated that SIRT1 was responsible for STAT3 deacetylation and, according with our results, Lovaas et al. [61] showed that SIRT1 induced an increase of *MMP2* gene expression, thus promoting tumor invasion. In parallel, other evidences, described the control of *SRD5A2* expression by inflammatory mediators (IL-6 and TNF-a) which upregulates DNMT1. The latter is responsible for the *SRD5A2* promoter methylation. This epigenetic event, that cause the inhibition of *SRD5A2* expression, is typically associated with the development of the hormone resistant form in prostate cancer. On the other hand, the occurrence of the pS^727^-STAT3 and the glutathionylated STAT3 forms [56, 68] as well as the increase of ERp57 protein suggests an oxidative stress environment in the higher Gleason Score tissues. In this scenario, the STAT3 PTMs profile and the presence and relative amount of its interactors in PCa might enable clinicians to make rational decisions concerning prognosis: the currently diagnosic elements, PSA levels, Gleason Score classification and TNM system, are not sufficient to predict which tumor will develop the aggressive form or stay indolent. For many years prostatectomy was the standard of care for PCa patients followed by a lower life quality. However the era of personalized medicine has arrived and is shaping therapy in several solid tumors. The need for individual treatment in prostate cancer is particularly notable because the disease is biologically and clinically heterogeneous. The correlation between STAT3 PTMs and tumor grades would be an additional biomarker for the exactly evaluation of the PCa stages and the optimal treatment options: Watchful Waiting for the indolent tumor, prostatectomy or hormone therapy for the aggressive ones [69]. In addition, because of their important role in cellular regulation, STAT3 PTMs might be important targets in cancer as adjuvants in chemiotherapy.

## MATERIALS AND METHODS

### Patients and tumor samples

Formalin fixed and paraffin-embedded (FFPE) blocks corresponding to PCa patients were retrieved from the archives of Pathology Laboratory “Department gynecological-obstetric and urological sciences” University “Sapienza” Rome, according to the following criteria: radical prostatectomy specimens with no previous treatment for PCa (including androgen deprivation therapy or chemotherapy prior to surgery). We obtained sixty-five PCa specimens during the period between 2008 and 2014, stratified in four groups by Gleason score. Patients and tissue data are summarized in Table 1. All patients gave written informed consent, and the study was approved by the Ethics Committee of our institution. Immunohistochemistry and Immunofluorescence staining

FFPE sections (5 μm), cut from each prostatectomy tissue block, were stained with hematoxylin/eosin and examined to localize normal and tumor sites (Supplementary Figure 1) [31]. Sections were subjected to immunofluorescence analysis [33]. Briefly, FFPE tumor and normal sections were deparaffinized in xylene and rehydrated through graded ethanol. The slides were subjected to heat-induced antigen retrieval in citrate buffer for 15 minutes. The sections were incubated with primary rabbit polyclonal antibody overnight at 4°C (p-Y705-STAT3 Cell Signaling Technology, diluited 1:200). After PBS washings, the incubation with secondary antibody FITC conjugated goat-anti-rabbit-IgG (Jackson Immunoresearch diluited 1:500) was performed for 1h at 25°C. The sections also were counterstained with propidium iodide (0, 3 μg/ml) for 10 minutes and mounted with glass coverslips. As negative controls, the sections, pretreated with 1% BSA, were incubated with secondary antibodies for 1h at room temperature. Additionally, primary antibodies were preincubated with a synthetic phosphopeptide (PpYLKTK, Sigma Aldrich) corresponding to residues flanking Tyr705 of STAT3 [33]. Stained sections were analyzed and photographed using a fluorescence microscope (Leica AF6000 Modular System) with 63× oil immersion objective. Scale bars 10 μm in magnifications.

### Protein extraction from FFPE sections

Normal and tumor FFPE sections (10 μm) were weighed (3 sections approximately equals to 30 mg) [34, 35]. The deparaffinization was carried out incubating twice with xylene for 5 minutes at room temperature before rehydration in a graded series of ethanol (100%, 90% and 70%) for 10 min each one. Vials containing the deparaffinized sections were put in a vacuum dryer for 15 minutes. Afterwards, samples were mixed with FFPE extraction buffer (150 μl per sample), sonicated about 7–8 times for 30 seconds, incubated at 100°C for 20 minutes and 80°C for 2 hours, and then centrifuged for 30 min at 14,000 rpm. The supernatant was supplemented with thiourea 100 mM and stored at −20°C. Protein concentrations were determined by the BCA assay (Bio-Rad, Munich, Germany).

### Extraction of total RNA from FFPE sections

Total RNA was extracted from three 10 μm thick FFPE sections (corresponding about 30 mg of tissue). RNA was purified with WAXFREE^™^ paraffin kit (TrimGen) according to the manufacturer's instructions. RNA was quantified spectrophotometrically, and its quality was assessed by 1.5% agarose gel electrophoresis and staining with ethidium bromide. To rule out the possibility of DNA contamination, the extracted RNA was incubated with 10 μm/ml RNase-free DNase at 37°C for 30 min.

### Reverse transcription and quantitative real time-PCR

The reverse transcription was carried out with PrimeScript^™^ RT reagent Kit (Takara). Gene expression was evaluated with specific primers for *PSA*, *BIRC5*, *SRD5A2*, *MMP2*, *CRP* and *SOD2* (Quanti-Tect QIAGEN) by Real-Time PCR using a MJ MiniOpticon Detection System (BioRad Laboratories) with SYBR green fluorophore using Brilliant SYBR Green QPCR Master Mix (Stratagene).

### Western blot analysis

Western blot analysis was carried out on proteins extracted from tumor and control regions of FFPE tissues (15–20 μg protein) resolved by 10% SDS-PAGE and transferred to nitrocellulose membranes [30]. The membranes were blocked with 3% nonfat dried milk in Tris-buffered saline containing 0.05% Tween-20 (TBS-T) and incubated with the desired primary antibody for 1h. Subsequently, the membranes were washed three times in T-TBS, and bound antibodies were detected using the appropriate horseradish peroxidase-conjugated secondary antibody (1 h) [37], followed by an ECL PlusWestern blotting Detection System (GE Healthcare Bio-Sciences). ECL was detected using a Molecular ImagerR ChemiDoc^™^ mod. MP System (Bio-Rad Laboratories), acquired by ImageLab Software ver. 4.1, and quantified using ImageJ analysis software (http://rsbweb.nih.gov/ij/). Measured protein abundances were normalized to actin amounts and the tumor/control abundance ratio for each protein was used for analysis. The ratios of tumor to control tissue values were used to correct for possible inter-individual differences in protein abundance not related to PCa. Immunodetection was carried out using polyclonal antibodies (Cell Signaling Technology; antibody dilution 1:1000) against STAT3 (cat. n. 9132), pY^705^-STAT3 (cat. n. 9131), pS^727^-STAT3 (cat. n. 9134) and acK^685^-STAT3 (cat. n. 2523). At least three experimental replicates were performed for each biological sample.

### Statistical analysis

The repeatability of results was confirmed by performing all experiments at least three times, and values are presented as the means, standard deviations (boxes) and min/max values (bars). Statistical analyses were performed with GraphPad software using a one-way ANOVA test followed by Bonferroni *post-hoc* test to analyze differences between Gleason groups.

## SUPPLEMENTARY FIGURES



## Abbreviations

PCa: Prostate Cancer
PSA: Prostate Specific Antigen
FFPE: Formalin Fixed and Paraffin Embedded
STAT3: Signal Transducer and Activator of Transcription
PTM: Post Translational Modification
pY^705^-STAT3: phosphorylation of tyrosine 705
pS^727^-STAT3: phosphorylation of serine 727
acK^685^-STAT3: acetylation of lysine 685
CBP/p300: CREB-binding protein/Histone Acetyltransferase p300 interactor
PDIA3/ERp57: Protein Disulfide-isomerase A3/Endoplasmic Reticulum Protein 57
APE1/Ref-1: Apurinic/apyrimidinic endonuclease 1/redox factor-1
FITC: Fluorescein isothiocyanate
TRITC: Tetramethylrhodamine
PI: Propidium Iodide
PBS: Phosphate Buffered Saline
*BIRC5*: Baculoviral Inhibitor of Apoptosis Repeat-containing 5
*SRD5A2*: Steroid-5-Alpha-Reductase, Alpha Polypeptide 2
MMP2: Matrix Metalloproteinase-2
*CRP*: C-reactive Protein
*SOD2*: Superoxide Dismutase 2
ROS: Reactive Oxygen Species
SIRT1: Sirtuin 1
DNMT1: DNA (cytosine-5)-methyltransferase 1
qRT-PCR: Quantitative Real Time - Polymerase Chain Reaction
SDS-PAGE: Sodium Dodecyl Sulphate - PolyAcrylamide Gel Electrophoresis
TBS: Tris-buffered Saline
ECL: Enhanced Chemiluminescence
