# Supplementary material for: Analysis of STAT3 post-translational modifications (PTMs) in human prostate cancer with different Gleason Score
Source: Oncotarget. 2017 Apr 19;8(26):42560–70. doi: 10.18632/oncotarget.17245 (PMC5522088; doi:10.18632/oncotarget.17245)
Supplement: Supplementary file 1 [file oncotarget-08-42560-s001.pdf]

Analysis of STAT3 post-translational modifications (PTMs) in human prostate cancer with different Gleason Score

Supplementary Materials

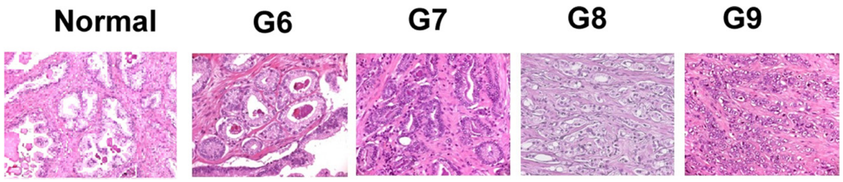

Supplementary Figure 1: Analysis of pY^{705}-STAT3 distribution in prostate normal FFPE tissues. Representative Hematoxylin and Eosin (H&E) staining of normal and different Gleason score FFPE tissue sections.

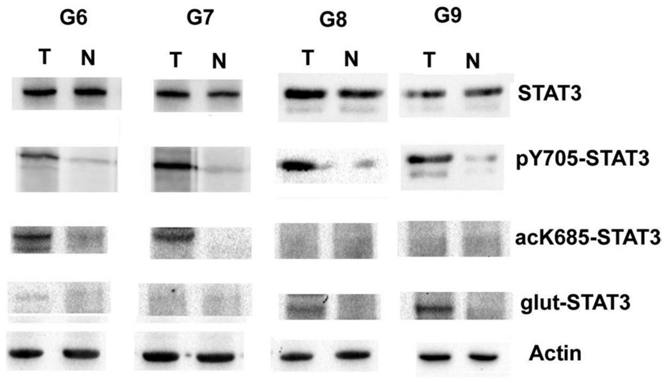

Supplementary Figure 2: Analysis of STAT3 PTMs in fresh tumor and normal tissues with different Gleason score. This image shows a representative western blot analysis of PTM's STAT3 in protein samples extracted from fresh tissues (biopsies). The proteins, as in FFPE tissues, were prepared from tumor and control regions of patients with Gleason score 6-7-8-9. This experiment was performed to confirm the efficiency of extraction from FFPE tissues. The results validate the efficiency and accuracy of the data obtained from the FFPE sections. T: prostate carcinomas; N: normal tissue. pY^{705}-STAT3: phosphorylated at tyrosine 705, acK^{685}-STAT3: acetylated at lysine 685, glu-STAT3: glutathionylated STAT3.

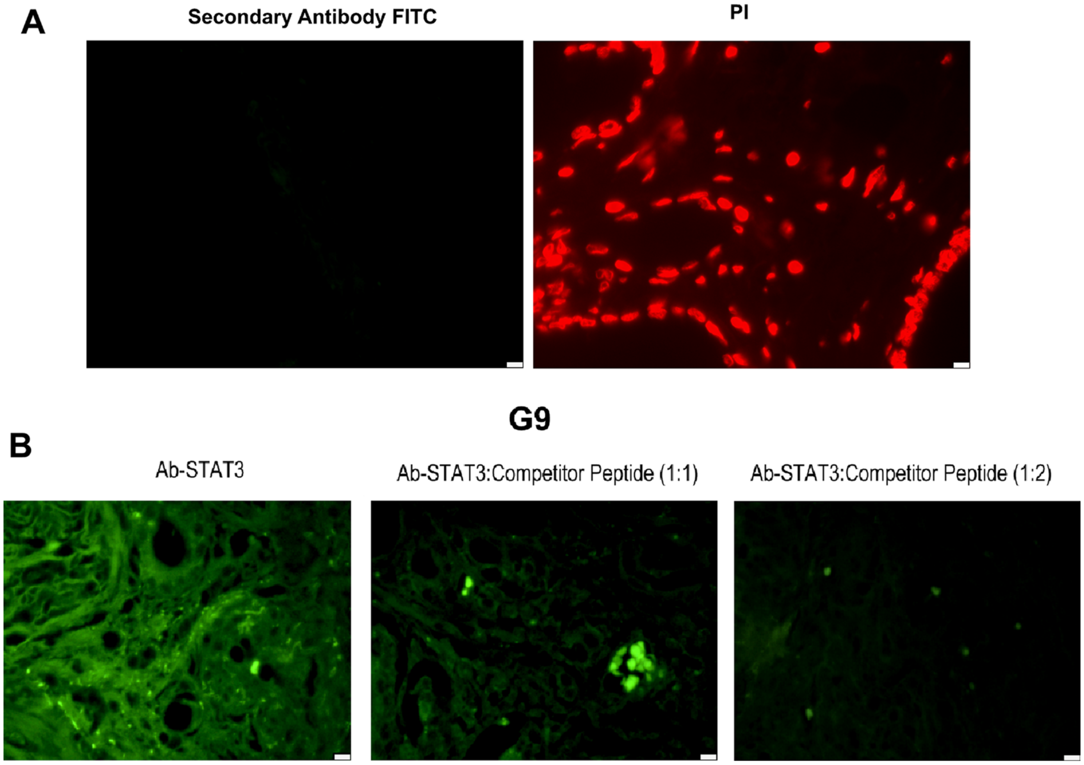

Supplementary Figure 3: Negative controls of immunofluorescence staining of FFPE tissue sections. Sections, pretreated with 1% BSA, were incubated with only secondary antibodies for 1 h at room temperature (**A**). Primary antibodies were preincubated with different amounts of a synthetic phosphopeptide (Sigma Aldrich) corresponding to residues flanking Tyr705 of STAT3 and then utilized for immunostaining (**B**). Stained sections were analyzed and photographed using a fluorescence microscope (Leica AF6000 Modular System) with 63 × oil immersion objective. Scale bars 10 µm in magnifications.
